# Early outdoor play predicts trajectories of child mental health in a population‐based cohort

**DOI:** 10.1111/jcpp.70175

**Published:** 2026-06-08

**Authors:** Helen F. Dodd, Kirsty Cordwell, Kathryn Hesketh, Avril Johnstone, Alejandro de la Torre‐Luque, Paul McCrorie

**Affiliations:** ^1^ Exeter Medical School University of Exeter Exeter UK; ^2^ Behavioural Science and Health Department University College London UK; ^3^ MRC/CSO Social and Public Health Sciences Unit University of Glasgow Glasgow UK; ^4^ Department of Legal Medicine, Psychiatry and Pathology, Faculty of Medicine Complutense University of Madrid Madrid Spain

**Keywords:** Outdoor play, trajectory, child mental health, internalising problems, externalising problems, prevention

## Abstract

**Background:**

Outdoor play may offer developmental opportunities that support good mental health. Cross‐sectional data indicate that more outdoor play is associated with better mental health, particularly internalising problems (e.g. anxiety and depression), but longitudinal research is lacking. We used a longitudinal cohort dataset to evaluate whether outdoor play at 2–4 years predicted children's mental health trajectories into middle childhood.

**Methods:**

Data were from the Growing up in Scotland dataset. Parents/caregivers were asked how many days their child had played outside over the previous week when children were aged ~2, 3 and 4 years. When the children were aged ~4, 5, 6 and 8 years, parents/caregivers completed the Strengths and Difficulties Questionnaire measure of child mental health. We used growth mixture modelling (GMM) to identify trajectories over time for internalising and externalising symptoms separately. Next, we used multilevel multinomial regression to evaluate whether early outdoor play predicted trajectory classification.

**Results:**

The analysis sample included 4,151 children. A three‐class solution was optimal for internalising and externalising symptoms. The three trajectory groups differed on both symptom level at age 4 and slope as follows: normative (low with stable trajectory); increasing (moderate with increasing trajectory); decreasing (high with decreasing trajectory). After controlling for covariates, outdoor play significantly predicted trajectory class; children who played outdoors more often as preschoolers had significantly decreased odds (*p* < .001) of belonging to the increasing or decreasing trajectory groups, relative to the normative group, for internalising (increasing: OR = 0.92, decreasing: OR = 0.88) and externalising (increasing: OR = 0.94, decreasing: OR = 0.94) symptoms.

**Conclusions:**

This study uses nationally representative longitudinal data and provides the first evidence that early outdoor play predicts mental health trajectories across childhood. A key limitation is reliance on parent‐report measures. The findings indicate that increasing outdoor play may be a useful public health approach to reduce child mental health problems.

## Introduction

Around the world, there is concern about children and young people's mental health (Global Mind Project, 2025). In Scotland an estimated 16% of children have an emotional or behavioural difficulty (Scottish Public Health Observatory, 2025), and similar rates have been reported in England, the United States, Canada, and Brazil (Centre for Disease Control, 2025; Marchionatti et al., 2024; NHS Digital, 2023; Statistics Canada, 2025). Many mental health problems, such as anxiety and depression, have their origins early in childhood (de Lijster et al., 2017; Kessler et al., 2005). Mental health problems during childhood have immediate consequences for young people's quality of life and opportunities (Celebre, Stewart, Theall, & Lapshina, 2021). Long‐term, if left untreated, mental health difficulties during childhood are related to a range of deleterious outcomes, such as reduced life satisfaction, poorer health‐related quality of life and lower academic attainment (Schlack, Peerenboom, Neuperdt, Junker, & Beyer, 2021), and have a substantial societal economic impact (Fatori et al., 2018; Future Minds, 2025). Identifying modifiable early life factors that may help to prevent the development of long‐term mental health problems is therefore critical for improving outcomes and guiding preventive interventions.

One modifiable early life factor is children's access to and engagement in outdoor play. Outdoor play refers to voluntary, intrinsically motivated activity that occurs in outdoor environments including gardens, playgrounds, natural spaces, and streets (Lee et al., 2022). It has been linked to a wide range of developmental outcomes, including social and cognitive development (Wyver, 2024) as well as emotional development, contributing to both emotion understanding and emotion regulation (Stavrou, 2019; Veiga, Sousa da Silva, Gibson, & Rieffe, 2022).

Whilst a wide range of factors influence the development of mental health problems in children, outdoor play may provide key learning experiences that provide a healthy foundation to prevent mental health difficulties, particularly internalising problems such as anxiety and depression, from developing (Whitebread, 2017). There are various mechanisms through which outdoor play may support good mental health including via physical activity (e.g. Barber et al., 2026) and connection with nature and place (Harris, 2021). Outdoor play also gives children opportunity to play adventurously, providing natural exposure to uncertainty and risk. Adventurous outdoor play may help children to develop a healthy understanding of physiological arousal, risk, uncertainty and effective coping mechanisms, that support the prevention of anxiety problems (Dodd & Lester, 2021). Further, outdoor play may provide exposure opportunities, such as exposure to heights, that help to prevent specific phobias (Sandseter & Kennair, 2011) and may facilitate the development of psychosocial competence more broadly (Hansen Sandseter, Kleppe, & Ottesen Kennair, 2023).

Despite its potential benefits, children's outdoor play has declined markedly in recent decades for a range of reasons, including increased road traffic, fears around safety and increased access to screen‐based activities (Lambert, Vlaar, Herrington, & Brussoni, 2019; Oliver, Nesbit, McCloy, Harvey, & Dodd, 2022, 2023). Access to outdoor play is unequal; even in the preschool years, girls and children belonging to racial and ethnic minority groups play outdoors less than their counterparts, especially in green spaces (Dodd & Hesketh, 2024). This decline in, and inequality in outdoor play may have important implications for children's health (Whitebread, 2017). Building on evidence that being outdoors, and in nature specifically, is good for children's health (Davis et al., 2021), more time spent playing outdoors specifically has been linked with lower internalising symptoms in preschool and school‐aged children (Dodd, Nesbit, & FitzGibbon, 2023; Hesketh & Dodd, 2026; Piccininni, Michaelson, Janssen, & Pickett, 2018). That said, most research that has explored these associations is cross‐sectional; across all health‐related outcomes fewer than 8% of papers published on outdoor play are longitudinal (De Lannoy, Barbeau, Seguin, & Tremblay, 2023). One of the few longitudinal studies to reference children's outdoor play opportunities reported that greater use of parks and playgrounds, and access to a garden, was associated with fewer behavioural problems across early childhood (Flouri, Midouhas, & Joshi, 2014).

To the best of our knowledge, despite the rapid social and emotional development that happens during early childhood (Sroufe, 2002), no research has examined associations between outdoor play during the preschool years and subsequent mental health. Given the need for early, preventative interventions for mental health (McDaid, Park, & Wahlbeck, 2019), investigating predictors in the preschool period is vital and outdoor play may offer a low‐cost, enjoyable, developmentally aligned opportunity to reduce children's risk for mental health difficulties. Many mental health disorders originate before adolescence, and experiencing mental health problems in early/middle childhood is linked to a greater risk of difficulties later in life (Mulraney et al., 2021). The aim of this study is therefore to evaluate whether outdoor play (defined as the number of days in a 1 week period that a child ran around or played outdoors) in the preschool years (ages 2, 3 and 4 years) is associated with trajectories of mental health through to middle childhood (ages 4–8 years), using data from a nationally representative cohort. We hypothesised that children with greater exposure to outdoor play in the preschool years would be more likely to follow favourable mental health trajectories into middle childhood.

## Method

### Participants

Data were from the ‘Growing Up in Scotland’ (GUS) dataset (Scottish Centre for Social Research, 2025), which initially followed 5,217 children born between June 2004 and May 2005 in Scotland. Details of the sampling framework are provided elsewhere (Bradshaw, Tipping, Marryat, & Corbett, 2005). GUS is a nationally representative, population‐based birth cohort following study participants from recruitment at 10 months. Data sweeps were conducted annually through to sweep 6 (~age 6), with sweep 7 conducted 2 years later, and weighted back to the population at each sweep. The current study uses data collected during sweeps 2–7, where the children were aged ~2 to ~8 years (see Table 1), with some demographic characteristics taken from sweep 1 data. Across all sweeps, data were reported by the primary caregiver who responded for themselves, their child and their partner, where appropriate. Detailed information about the dataset, measures, and approach is available online (https://growingupinscotland.org.uk/about‐gus).

**Table 1:** Descriptive data by sample in analysis and attrition analysis

	*n*	Response rate^a^	*m* Age (months)	SD (months)	Range
Sweep 2	4,512	86.5	22.16	0.44	19–24
Sweep 3	4,193	92.9	34.6	0.44	33–36
Sweep 4	3,994	95.3	46.18	0.48	45–48
Sweep 5	3,833	96.0	58.17	0.45	57–60
Sweep 6	3,657	95.4	70.24	0.48	69–72
Sweep 8	3,456	94.5	94.41	0.76	83–100

Variable	Initial sample (*N* = 4,512)		Sample in symptom trajectory analysis (*n* = 4,151)		Sample in regression analysis (*n* = 4,151)		Attrition analysis (sample in symptom trajectory analysis vs. excluded)		Attrition analysis (sample with complete data for regression analysis (3080) vs. missing)	
	*M*/%	*SD*	*M*/%	*SD*	*M*/%	*SD*	*χ* ^2^/*t*	ES	*χ* ^2^/*t*	ES
Deprivation index							38.71**	0.09	431.8**	0.29
1st quintile (least deprived)	20.5		21.1		25.4					
2n quintile	21.1		21.3		23.2					
3rd quintile	20.5		20.8		21.7					
4th quintile	17		17.1		15.7					
5th quintile (most deprived)	20.9		19.7		14.1					
Change in Deprivation index quintile from sweep 2 to sweep 3 (%no)	89.4		89.4		89.9		0.08	0.01	4.39*	0.03
Child Ethnicity (White Caucasian)	95.5		96.2		96.2		24.52**	0.07	10.39**	0.05
Child Sex (Male)	51.2		51.1		50.8		0.01	0	0.64	0.01
Level of education of at least one parents							3.36	0.03	1.75	0.02
Higher or above	67.2		67.4		67.4					
Standard grade or other	21.6		21.6		21.4					
No qualifications	11.2		11		11.1					
Parent working status (both parents not working)	13.8		12.1		4.7		95**	0.14	780.01**	0.39
Access to a park within 10 min (%yes)	86.7		86.7		86.2		0	0	1.66	0.02
Access to a garden (%yes)	88.8		90.9		93.5		89.94**	0.13	181.94**	0.19
Number of physical conditions	2.05	2.36	2.09	2.4	2.06	2.36	2.1*	0.07	0.35	0.01
Caregiver mental health	49.71	10.24	49.91	9.76	50.44	9.18	2.47*	0.10	6.18**	0.19
Outdoor play (number of days/week)										
Sweep 2	5	2.5	5.02	2.49	5.06	2.46	1.57	0.08	2.56*	0.09
Sweep 3	5.06	2.35	5.09	2.34	5.12	2.31	2.67**	0.21	2.72**	0.1
Sweep 4	5.17	2.25	5.18	2.25	5.22	2.21	3.2*	1.64	2.51*	0.1
Internalising symptoms										
Sweep 4	2.28	2.28	2.27	2.28	2.11	2.12			−7.35**	−0.3
Sweep 5	2.23	2.3	2.23	2.3	2.07	2.15			−6.77**	−0.28
Sweep 6	2.16	2.42	2.16	2.42	1.98	2.26			−7.13**	−0.32
Sweep 7	2.56	2.82	2.56	2.82	2.37	2.63			−6.47**	−0.3
Externalising symptoms										
Sweep 4	5.47	3.12	5.47	3.12	5.18	2.99			−9.71**	−0.37
Sweep 5	5.31	3.21	5.31	3.21	5.02	3.09			−9.53**	−0.38
Sweep 6	4.99	3.31	4.99	3.31	4.69	3.18			−9.8**	−0.41
Sweep 7	4.79	3.49	4.79	3.49	4.5	3.34			−8.6**	−0.38

Percentage of cases are displayed for categorical data and mean and standard deviation are displayed for continuous data. Data displayed did not include imputed values. The *χ* ^2^ test and Cramer's *V* statistic (effect size) were used for categorical data comparison. The Student's *t*‐test and Cohen's *d* statistic (effect size) were used for continuous data comparison. ES, effect size estimate. *p < .05; **p < .01.

^a^ Response rate in comparison to baseline (Sweep 2)/previous sweep (Sweeps 3–7).

### Measures

#### Exposure

Outdoor play was assessed at age 2, 3, and 4 years by asking parents, ‘How many days in the last week [7 days] has [child's name] run around or played outdoors?’ The response was then coded 0–7 for the number of days in the week and used as a continuous variable (see the Figure S1).

#### Outcomes

Mental health outcomes (externalising problems and internalising symptoms) were assessed via parent report using the Strengths and Difficulties Questionnaire (SDQ) (Goodman, 2011), a 25‐item questionnaire with five subscales: emotional symptoms, conduct problems, hyperactivity–inattention, peer problems, and prosocial behaviour. The internalising problems scale includes emotional symptoms and peer problems subscales, and the externalising symptoms scale includes conduct problems and hyperactivity–inattention subscales. The parent‐report SDQ is well‐validated for use with younger children who may not yet be able to provide reliable self‐report; it is psychometrically sound and is used to provide the national statistics on children's mental health in England (NHS Digital, 2023). The use of the internalising and externalising scales is deemed most appropriate for low‐risk epidemiological samples (Goodman, Lamping, & Ploubidis, 2010). The SDQ was conducted at sweeps 4, 5, 6, and 7, and internalising (Cronbach's alpha between .64 at sweep 4 and .78 at sweep 5) and externalising scores (Cronbach's alpha between .67 at sweep 4 and .75 at sweep 7) were calculated.

#### Covariates

A range of demographic factors were included as covariates, selected from the available data and identified as potential confounders using a directed acyclic graph (DAG; see Figure S2). Included covariates collected at sweep 1 were: child sex (male/female); ethnicity of child (White/Not white); highest education level within household (reporting caregiver or partner) (defined in three categories of: higher and above; standard grade/other; no qualification); and number of physical conditions that the child has. From sweep 2 (our baseline), the Scottish index of multiple deprivation (SIMD) was derived, encompassing area level deprivation across income, employment, education, health, access to services, crime and housing, and presented as quintiles (Scottish Index of Multiple Deprivation 2020). We also derived an additional variable to capture any change in SIMD from sweep 2–3. Working status of parent and partner was also derived (binary, neither parent working/at least one parent employed). In addition, binary (yes/no) responses regarding whether the family had access to a park within 10 min of home and access to a garden were included. These were assessed at sweep 1 and updated at sweeps 2 and 3 if the child had moved. We also included caregiver mental health, which was assessed using the SF‐12 Mental Component Summary (Ware, Kosinski, & Keller, 1996), as 12‐item self‐report scale used extensively as a screening tool for overall mental health.

### Analysis plan

Mean and standard deviation estimates were calculated from continuous variables and proportion of cases for categorical data. A two‐step approach was taken to the analysis. First, growth mixture modelling (GMM) was used to identify heterogeneous developmental trajectories over time for internalising and externalising symptom trajectories separately (Berlin, Parra, & Williams, 2014). Trajectory enumeration was based on unconstrained GMM models without any time‐invariant nor time‐dependent covariates that may capture the outcome variance, to decrease the probability of class overestimation (Vermunt, 2010). Second, multilevel multinomial regression was used to evaluate the extent to which outdoor play, as assessed at sweeps 2, 3, and 4 predicted trajectory classification. The overall trend in trajectories of internalising and externalising scales is shown on Figure S1.

For the GMM analysis, growth parameters were estimated using linear, quadratic, and cubic polynomial time terms (age in months, centred at zero). Missing data were handled using full information maximum likelihood on the endogenous data level (i.e. on the predicted values of the dependent variable), allowing participants with missing data to be included in the analysis (Cham, Reshetnyak, Rosenfeld, & Breitbart, 2017). GMM solutions with the optimal class enumeration were selected based on the comparison model tradition. In this regard, models with increasing trajectory classes were tested. Criteria to select the model with the optimal class enumeration were: (i) low sample‐adjusted Bayesian information criterion (SABIC) and Akaike information criterion (AIC), (ii) mean of posterior probabilities to belong to each identified class higher than .80; and (iii) meaningful proportion of participants within each class (>5%).

Multinomial multilevel regression analyses were then used to estimate odds ratio (OR) estimates for association between outdoor play and trajectory membership, using the normative groups as the baseline comparator for both outcome analyses. The multinomial multilevel model incorporated frequency of outdoor play as a time‐varying predictor at Level 1, nested within children (Level 2), with each child contributing up to three observations (sweeps 2, 3, 4). This multilevel specification was used to account for the clustering of repeated observations within children, providing child‐level estimates of early outdoor play patterns, to be used as predictors of trajectory class membership. Analyses were conducted for internalising and externalising trajectories separately, with trajectory class membership of the opposed symptom domain (internalising for externalising and vice versa) included as a covariate. The ORs indicate the relative odds of being classified into each trajectory group, compared with the normative trajectory group, per unit increase in outdoor play. Thus, an OR greater than 1 indicates higher outdoor play is associated with increased odds of being in that trajectory group (vs. normative), an OR less than 1 indicates decreased odds of being in that trajectory group (and increased odds of being in the normative group). The same sociodemographic and clinical covariates (see above) were included for both outcomes and the GUS sweep 4 longitudinal weights were used as weighting variables to control for longitudinal attrition.

All analyses were conducted using R (R Core Team, 2021; packages lcmm, mice, nnet).

## Results

Data were available at sweep 2 for 4,512 participants: 4151 of these had SDQ data available at two or more sweeps across 4, 5, 6, and 7 and therefore formed our analysis sample. Demographic characteristics of this sample and attrition analysis are presented in Table 1. The analysis sample, included in the GMM analyses, and the dropout sample only differed notably on outdoor play, which was less common at sweeps 3 and 4 in the dropout sample.

For the regression analyses, 3,080 had complete data before imputation (see Table 1 and Table S1 for covariate breakdown). Differences with at least a small effect size (ES > 0.2) were observed between those with complete data and those with missing data on SIMD, parent working status, internalising and externalising symptoms. Missing values were estimated using multiple imputation via the mice package, under the random forest algorithm, with 50 iterations and 10 rounds of imputation. The distributions of the original and imputed data are displayed in Figure S3; no auxiliary variables were used.

### Step 1: GMM

The GMM analysis indicated that a three‐class solution was optimal for both the internalising symptom score (with quadratic shape) and the externalising symptom score (with cubic shape) (see Figure 1). Model comparison indexes for both outcomes are displayed in Table S2. In terms of internalising symptoms, of the three trajectories identified, most cohort members (87%, *n* = 3,610) showed a course of minimal internalising symptoms (i.e. normative). Over 8% of children (*n* = 324) showed a trajectory of increasing symptoms over time, with 5% (*n* = 217) showing a trajectory of decreasing symptoms over time. Symptoms were consistently elevated between ages 4 and 8 relative to the normative group in both the non‐normative (increasing and decreasing) groups. For externalising symptoms, most children showed a course of minimal symptoms over childhood (82%, *n* = 3,387). Around 13% of participants (*n* = 526) displayed an increase in externalising symptoms over time, whereas 6% of participants (*n* = 236) showed a trajectory of decreasing symptoms (See Table 2 for GMM model parameters). Again, symptoms were consistently elevated between ages 4 and 8 in both the non‐normative (increasing and decreasing) groups relative to the normative group.

**Figure 1:**
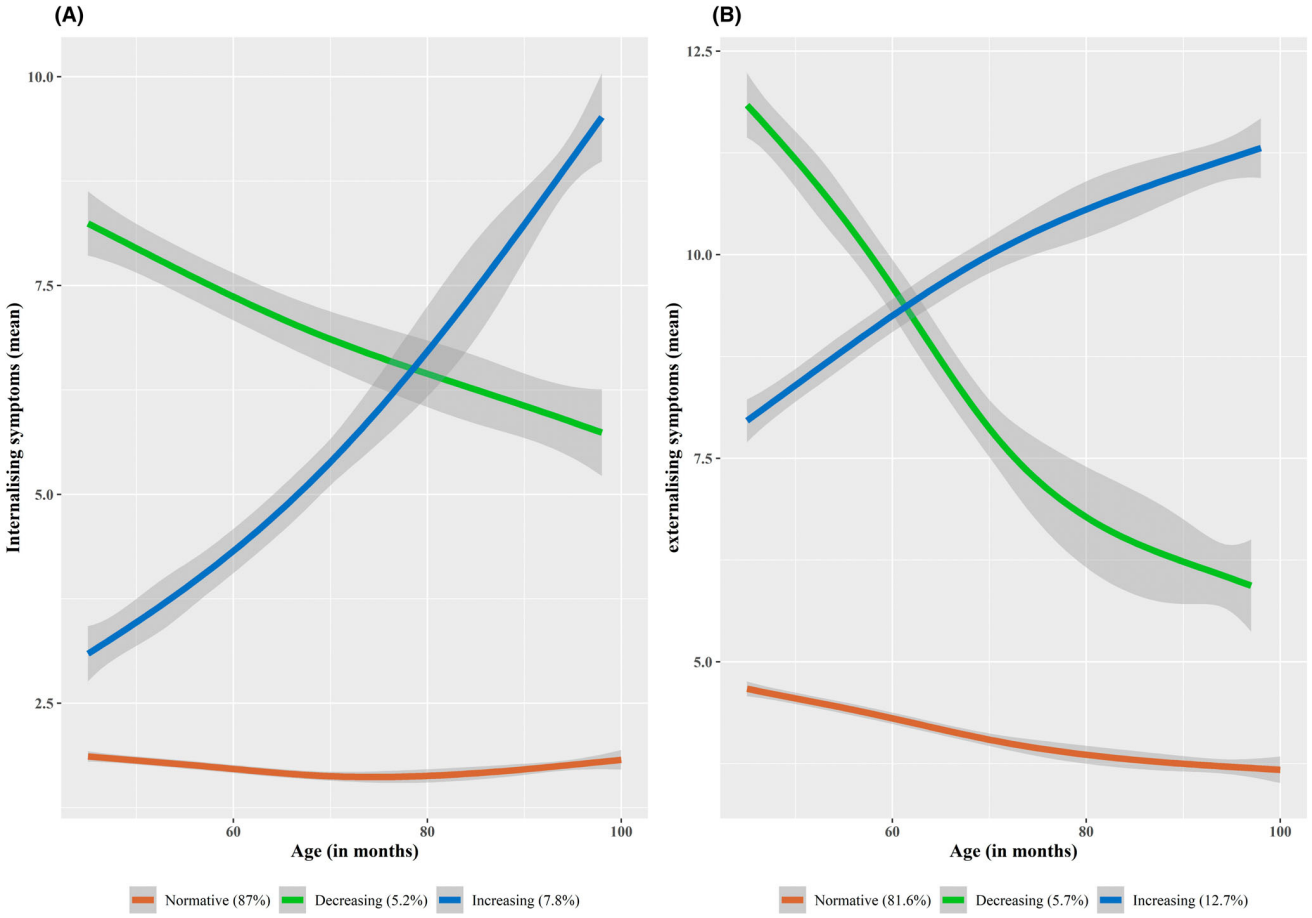
Predicted trajectories of internalising and externalising symptoms by age, split by trajectory class

**Table 2:** GMM model parameter for the SDQ outcomes

	Internalising symptom model			Externalising symptom model		
	*B*	*SE*	*Z*	*B*	*SE*	*Z*
Class = Normative						
Intercept	1.87	0.04	49.58**	4.62	0.08	59.33**
Time (Linear term)	−0.02	0.00	−6.25**	−0.01	0.01	−0.81
Time (Quadratic term)	0.00	0.00	5.89**	0.00	0.00	−1.85
Time (Cubic term)^a^				0.00	0.00	2.30*
Class = Decreasing						
Intercept	7.97	0.23	35.40**	10.38	0.35	29.55**
Time (Linear term)	−0.07	0.02	−3.77**	−0.02	0.05	−0.43
Time (Quadratic term)	0.00	0.00	1.26	−0.01	0.00	−2.49*
Time (Cubic term)^a^				0.00	0.00	2.95**
Class = Increasing						
Intercept	3.06	0.14	22.04**	7.57	0.18	42.92**
Time (Linear term)	0.06	0.01	4.89**	0.07	0.03	2.48*
Time (Quadratic term)	0.00	0.00	5.54**	0.00	0.00	0.27
Time (Cubic term)^a^				0.00	0.00	−0.61
Random‐effect intercept (overall model)	1.39	1.45		3.89	1.7	

*B*, estimated loading; *SE*, standard error of the loading; *Z*, Wald's *Z* test. *p < .05; **p < .01.

^a^ Only included in the externalising symptom model.

Regarding overlap in membership of internalising and externalising trajectory groups, 25% of our sample were classified as belonging to a ‘non‐normative’ trajectory group in at least one domain (internalising or externalising) but only 7% were in a ‘non‐normative’ trajectory group for *both* internalising and externalising symptoms, and <2% were categorised to the corresponding non‐normative trajectory groups (increasing/decreasing) across internalising and externalising domains. A full breakdown of classification overlap is provided in Figure S4.

### Step 2: Multilevel multinomial logistic regression

The results are shown in Tables 3 and 4 respectively. For internalising symptoms, after controlling for covariates, outdoor play was a significant predictor of trajectory class membership: children who played outdoors more often as preschoolers were less likely to belong to both the increasing and decreasing internalising symptom trajectory groups than those who played out less often. For externalising problems, after controlling for covariates, children's outdoor play across sweeps 2, 3, and 4 significantly predicted trajectory group membership: children who played outdoors more often were less likely to belong to the increasing or decreasing externalising symptom trajectory group when compared with those who played outdoor less often. Note that we also conducted a comparable multilevel binary logistic regression to examine whether outdoor play was a predictor of increasing versus decreasing trajectory classes. For externalising symptoms, outdoor play was not a significant predictor. On the other hand, outdoor play was associated with a higher probability of being a member of the increasing internalising symptom trajectory group in comparison to the decreasing trajectory group, OR = 1.13, *p* < .05 (see Table S3).

**Table 3:** Relationship between the internalising symptom trajectory membership and covariates

	Decreasing trajectory class				Increasing trajectory class			
	OR	LB (CI_95_)	UB (CI_95_)	*Z*	OR	LB (CI_95_)	UB (CI_95_)	*Z*
Outdoor play	0.88	0.84	0.92	−5.34**	0.93	0.89	0.97	−3.51**
Deprivation index (ref.: 1st quintile)								
2nd quintile	1.52	1.22	1.89	3.76**	1.35	1.15	1.57	3.72**
3rd quintile	2.05	1.67	2.52	6.91**	1.24	1.06	1.45	2.73*
4th quintile	2.55	2.09	3.13	9.08**	1.45	1.24	1.7	4.66**
5th quintile	2.38	1.94	2.91	8.4**	1.65	1.41	1.92	6.39**
Change in deprivation quintile^a^	0.78	0.65	0.93	−2.75*	1.13	0.99	1.29	1.84
Ethnicity (ref.: White Caucasian)	2.71	2.26	3.24	10.81**	0.8	0.63	1.02	−1.82
Child sex (ref.: Male)	0.67	0.6	0.75	−7.26**	1.17	1.07	1.27	3.44**
Maximum level of education of at least one parent (ref.: Higher or above)								
Standard grade or other	1.26	1.12	1.43	3.7**	1.27	1.15	1.41	4.56**
No qualifications	1.07	0.93	1.24	0.97	1.09	0.96	1.24	1.4
Parent working status (ref.: parents not working)	0.73	0.64	0.84	−4.6**	0.6	0.53	0.67	−8.92**
Respondent's mental health	0.79	0.76	0.82	−11.62**	0.79	0.76	0.82	−12.97**
Access to a park within 10 min (ref.: No)	1.23	1.05	1.45	2.5*	0.99	0.88	1.12	−0.12
Access to a garden (ref.: No)	0.87	0.75	1	−1.96	0.77	0.68	0.87	−4.18**
Child health problems	1.1	1.06	1.14	5.08**	1.13	1.09	1.17	6.88**
Internalising symptom trajectory class (ref.: normative)								
Decreasing trajectory	6.96	6.09	7.95	28.5**	1.38	1.14	1.65	3.38**
Increasing trajectory	4.28	3.77	4.85	22.78**	7.38	6.72	8.1	41.9**

The outcome is the membership to an internalising symptom trajectory class, with reference category = normative class membership. This regression model fitted better to data with Akaike information criterion (AIC) = 27069.55 (AIC for the unconstrained model = 31639.64). The model included the longitudinal weights for the cohort as a weighting variable. LB (CI_95_), 95% Confidence interval of the OR (lower bound); OR, Odds ratio; UB (CI_95_), 95% Confidence interval of the OR (upper bound); *Z*, Wald's test statistic. * *p* < .05. ** *p* < .01.

^a^ Binary variable capturing whether quintile changed between sweeps 2 and 3.

**Table 4:** Relationship between the externalising symptom trajectory membership and covariates

	Decreasing trajectory class				Increasing trajectory class			
	OR	LB (CI_95_)	UB (CI_95_)	*Z*	OR	LB (CI_95_)	UB (CI_95_)	*Z*
Outdoor play	0.95	0.9	0.99	−2.33*	0.94	0.91	0.98	−3.34**
Deprivation index (ref.: 1st quintile)								
2nd quintile	1.13	0.94	1.34	1.3	0.95	0.83	1.07	−0.87
3rd quintile	1.25	1.05	1.49	2.5*	1.33	1.18	1.5	4.68**
4th quintile	1.15	0.96	1.37	1.5	0.94	0.82	1.06	−1.01
5th quintile	1.41	1.19	1.68	3.92**	1.31	1.16	1.48	4.36**
Changes in deprivation quintile from sweep 2 to sweep3	0.96	0.82	1.12	−0.55	0.93	0.83	1.04	−1.28
Ethnicity (ref.: White Caucasian)	1.79	1.48	2.17	5.97**	1.07	0.9	1.27	0.74
Child sex (ref.: Male)	0.84	0.76	0.92	−3.55**	0.41	0.38	0.44	−23.35**
Maximum level of education of at least one parent (ref.: Higher or above)								
Standard grade or other	1.45	1.29	1.63	6.29**	1.42	1.3	1.55	7.9**
No qualifications	1.27	1.1	1.46	3.33**	1.64	1.48	1.81	9.45**
Parent working status (ref.: parents not working)	0.47	0.41	0.53	−11.83**	0.64	0.58	0.71	−8.78**
Respondent's mental health	0.81	0.78	0.84	−10.38**	0.86	0.83	0.89	−9.46**
Access to a park within 10 min (ref.: No)	0.97	0.84	1.12	−0.45	0.99	0.9	1.1	−0.1
Access to a garden (ref.: No)	0.64	0.56	0.73	−6.46**	0.72	0.65	0.8	−6.12**
Child health problems	1.24	1.19	1.28	12.28**	1.02	0.99	1.06	1.17
Internalising symptom trajectory class (ref.: normative)								
Decreasing trajectory	6.9	6.03	7.88	28.24**	4.22	3.72	4.78	22.47**
Increasing trajectory	1.38	1.15	1.66	3.4**	7.31	6.66	8.04	41.51**

The outcome is the membership to an externalising symptom trajectory class, with reference category = normative class membership. This regression model fitted better to data with Akaike information criterion (AIC) = 34079.22 (AIC for the unconstrained model = 39424.45). The model included the longitudinal cohort weights as a weighting variable. LB (CI_95_), 95% Confidence interval of the OR (lower bound); OR, odds ratio; UB (CI_95_), 95% Confidence interval of the OR (upper bound); *Z*, Wald's test statistic. *p < .05; **p < .01.

## Discussion

This study investigated whether outdoor play during the preschool years predicts mental health symptom trajectories through to middle childhood in a large, nationally representative cohort. Consistent with our hypothesis, we found that greater frequency of outdoor play between ages 2 and 4 years was associated with an increased likelihood of belonging to the healthy trajectory category for internalising and externalising problems up to age 8. Children who played outdoors more frequently were more likely to follow a trajectory of minimal symptoms, demonstrating good mental health over time.

We took a two‐stage approach to the analysis, first identifying symptom trajectory groups for both internalising and externalising domains. The three distinct trajectories identified (normative, increasing, and decreasing) align with previous longitudinal research exploring trajectories of internalising and externalising problems over time in children of a similar age (Parkes, Sweeting, & Wight, 2016; Yoon, 2017). Other work suggests that, in samples that include older children (up to and including age 11), more trajectory classes may be necessary to accurately capture mental health outcomes through to adolescence (Papachristou & Flouri, 2020; Patalay, Deighton, Fonagy, & Wolpert, 2015).

The second stage of our analysis focused on outdoor play as a predictor of trajectory. Clearly, a wide range of factors affect children's mental health, but outdoor play may facilitate the development of cognitive, social, and emotional skills that help to prevent poor mental health. Whilst previous research has demonstrated cross‐sectional associations between children's outdoor play and their mental health (Dodd et al., 2023; Piccininni et al., 2018), this is the first study to demonstrate that outdoor play during the preschool years is prospectively associated with children's trajectories of mental health. The results clearly show that preschool children who play outdoors more frequently have better mental health through to middle childhood; more outdoor play during the preschool years significantly predicted membership of the normative trajectory category, relative to the increasing and decreasing categories. This result was consistent across internalising and externalising domains. In addition, more play also increased the odds of a child belonging to the increasing trajectory of internalising symptoms relative to a decreasing trajectory. Previous research has found associations between play and internalising, but not externalising symptoms (Dodd et al., 2023; Hesketh & Dodd, 2026), and relevant theory focuses on internalising problems like anxiety. The significant associations between play and externalising problems are therefore noteworthy. The research aligns with the established literature demonstrating that early life experiences are associated with mental health across childhood, and it significantly contributes to understanding of how outdoor play relates to child mental health.

As outlined, there are several plausible mechanisms through which outdoor play during the preschool years may affect children's mental health. Outdoor play supports exposure to and connection with nature, physical activity, and social interaction, as well as opportunities for adventurous and risky play, all of which have been shown to provide mental health benefits via diverse pathways (Dodd et al., 2023; Dodd & Hesketh, 2024; Dodd & Lester, 2021; Flouri et al., 2014; Piccininni et al., 2018; UK Government, 2019; Whitebread, 2017). With a growing body of research demonstrating associations between outdoor play and child mental health, this work suggests a plausible longitudinal relationship, and provides a foundation for future research to address key questions regarding underpinning mechanism and intervention opportunities.

### Strengths and limitations

Whilst our findings support directional inference, we cannot conclude that outdoor play is causally related to children's mental health. The longitudinal design is a strength but to move this field forwards and assess causality, it will be necessary to conduct interventions that increase children's outdoor play within an experimental design, and evaluate the impact of those changes in play on children's mental health. The barriers to doing this are significant; it relies on an effective intervention to increase outdoor play, outcomes may not be apparent until later in development, and effects are likely to be small, although noteworthy at a population level (Cuijpers, 2003; McDaid et al., 2019). Longitudinal observational designs such as that employed in the present study therefore provide vital insights.

We were able to control for a range of confounding variables and show that the associations of interest held after controlling for them. It is a strength of the dataset that a broad range of demographic factors were assessed, but child temperament and parenting style were identified as possible confounders in the DAG, but were not available in the dataset extract that we had access to. Also, data for ethnicity was only available as a binary category, which does not align with guidance for reporting of race and ethnicity (Flanagin et al., 2021). For context, around 96% of those living in Scotland identify as white (Scotland's Census, 2022); the data regarding specific ethnic categories in the GUS dataset therefore presents a disclosure risk when combined with the other demographic data, meaning that only the binary version of the variable is available for use. The lack of racial and ethnic diversity in the cohort impacts the transferability of the study's conclusions to diverse racial and ethnic groups.

A further key limitation is that outdoor play was only assessed via a parent report question. Since the data were collected, new survey measures of outdoor play have been developed that would provide much more nuanced insights into the types, timings and locations of outdoor play (Dodd, Nesbit, & Maratchi, 2021). Other approaches that would be more methodologically robust include time‐use diaries and objective measures such as GPS and GIS, although there are challenges to using these with large cohorts (Loebach & Gilliland, 2016). Future longitudinal work would benefit from a more detailed analysis of early outdoor play, using multiple methods. Given that parent report was also used for the mental health outcome measure, associations could be driven by social desirability bias. Some design features reduce the likelihood of social desirable responding. For example, participants could not have known our hypotheses when participating, data were collected confidentially and largely anonymously, standardised measures were used where available and there were no incentives to respond in a socially desirable way. Further, it is not clear that social desirability bias would yield the three trajectory groups, which align with previous longitudinal research, nor differential associations between outdoor play and the increasing and decreasing groups. Nevertheless, we recognise that social desirability bias could account for some of the association between outdoor play and membership of the normative trajectory group, and that future research using intervention, observational or objective measures would strengthen causal inference.

### Implications

The findings have implications for public health approaches to preventing child mental health problems. Rapid social and emotional development happens during the preschool years, yet children's outdoor play is declining, and inequalities in access to outdoor play opportunities are evident (Dodd & Hesketh, 2024). Supporting outdoor play during the preschool years requires contextually situated multilevel interventions focusing on areas of most need, for example, community‐based initiatives, guidance for childcare settings about providing outdoor play, support for parents, funding for high‐quality play and green spaces, and urban design and planning processes that support and encourage equitable access. Combined with targeted and sustained public health messaging, these may provide a low‐cost, scalable, and enjoyable way to build a positive foundation for children's mental health. A focus on equitable access and reduction of inequalities is key to ensuring that outdoor play interventions reduce inequalities in both play and health.

## Conclusion

This study provides novel evidence that greater engagement in outdoor play during the preschool years is associated with more favourable trajectories of internalising and externalising symptoms into middle childhood. Underpinned by robust, nationally representative cohort data, our longitudinal research provides a valuable addition to the literature. Given rising concerns about children's mental health and declining play opportunities, these findings highlight the need to incorporate outdoor play into the preventative public mental health agenda.

## Ethical considerations

This secondary data analysis was reviewed and approved by the University of Exeter Medical School Research Ethics Committee (Application reference: 5412698, approved 29/07/2024). Details of the original ethical approval and consent procedures for the Growing up in Scotland cohort are available at: https://growingupinscotland.org.uk/study‐design‐and‐timeline.

**Key points**

**What's known?**

- Cross‐sectional research shows that outdoor play is associated with better mental health in children.

**What's new?**

- This is the first long‐term longitudinal study examining associations between outdoor play and subsequent mental health.
- Using a cohort dataset, we found that children who played outdoors more often at ages 2–4 years were more likely to have a healthy trajectory of mental health symptoms through to middle childhood.

**What's relevant?**

- The findings have relevance for public health policy and educational practice in relation to providing for and protecting children's outdoor play opportunities.

## Supporting information


**Table S1.** Number of missing values and percentage of missing values per outcome and covariates.
**Table S2**. GMM fit indexes for the SDQ outcomes.
**Table S3**. Relationship between the symptom trajectory membership and covariates, for the non‐normative trajectory groups only.
**Figure S1**. Average trajectory of SDQ internalising (left side) and externalising (right side) symptoms across childhood measurement points.
**Figure S2**. Directed acyclic graph (DAG).
**Figure S3**. Density plot on the original distribution of variables (blue ink) with imputed data. The distribution of imputed data are displayed according to the different round of iterations (in red ink).
**Figure S4**. Proportion of sample belonging to each trajectory category across internalising and externalising domains.

## Data Availability

The data that support the findings of this study are openly available in UK Data Service at: https://doi.org/10.5255/UKDA‐SERIES‐200020.
