# Supplementary material for: Early outdoor play predicts trajectories of child mental health in a population‐based cohort
Source: J Child Psychol Psychiatry. 2026 Jun 8;67(11):1820–30. doi: 10.1111/jcpp.70175 (PMC13647091; doi:10.1111/jcpp.70175)
Supplement: Supplementary file 1 [file JCPP-67-1820-s001.docx]

# **Early Outdoor Play Predicts Trajectories of Child Mental Health in a Population-Based Cohort**

**Supporting Information**

**Table S1. Number of missing values and percentage of missing values per outcome and covariates.**

| Variable in analysis | Number of missing values | Percentage of data missing |
| --- | --- | --- |
| Outdoor play (all sweeps in analysis) | 402 | 3.2 |
| Deprivation index (sweep 2) | 118 | 2.8 |
| Change in Deprivation index from sweep 2 to sweep 3) | 238 | 5.7 |
| Ethnicity | 2 | 0 |
| Child sex | 149 | 3.6 |
| Maximum level of education of at least one parent | 952 | 22.9 |
| Parent working status (ref.: parents not working) | 107 | 2.6 |
| Access to a park within 10 minutes (sweep 2) | 152 | 3.7 |
| Access to a garden (sweep 2) | 7 | 0.2 |
| Child health problems | 2 | 0 |
| Externalising symptom trajectory class | 2 | 0 |
| Internalising symptom trajectory class | 0 | 0 |
| Respondent Mental health (sweep 1) | 0 | 0 |
| Birth cohort longitudinal weight | 161 | 3.9 |

***Note.*** Imputation was conducted on the longitudinal dataset. The percentage of missing values was calculated dividing the total number of missing values by total number of data points for that variable in the regression analysis (data from 4151 participants), note that for the time invariant outdoor play variable the total number of data points was 4151 across 3 waves (12453).

**Table S2. GMM fit indexes for the SDQ outcomes.**

|  | Class | Time effect shape | BIC | SABIC | AIC | min% per class | max% per class |
| --- | --- | --- | --- | --- | --- | --- | --- |
| Internalising symptom models | 1 | No time effect | 63654.40 | 63644.87 | 63635.41 | 100 |  |
|  | 1 | Linear | 63592.38 | 63579.67 | 63567.05 | 100 |  |
|  | 1 | Quadratic | 63542.52 | 63526.63 | 63510.87 | 100 |  |
|  | 1 | Cubic | 63548.60 | 63529.54 | 63510.62 | 100 |  |
|  | 2 | No time effect | 63671.07 | 63655.18 | 63639.41 | 38.95 | 61.05 |
|  | 2 | Linear | 62167.19 | 62144.94 | 62122.87 | 8.07 | 91.93 |
|  | 2 | Quadratic | 62100.00 | 62071.40 | 62043.02 | 8.05 | 91.95 |
|  | 2 | Cubic | 62113.66 | 62078.70 | 62044.01 | 8.00 | 92.00 |
|  | 3 | No time effect | 62771.04 | 62748.79 | 62726.72 | 0.00 | 88.99 |
|  | 3 | Linear | 61429.00 | 61397.22 | 61365.69 | 5.32 | 86.89 |
|  | **3** | **Quadratic** | **61369.81** | **61328.50** | **61287.51** | **5.23** | **86.97** |
|  | 3 | Cubic | 61387.08 | 61336.24 | 61285.79 | 5.08 | 87.11 |
|  | 4 | No time effect | 62314.72 | 62286.12 | 62257.74 | 0.00 | 72.99 |
|  | 4 | Linear | 61453.99 | 61412.68 | 61371.69 | 0.00 | 84.82 |
|  | 4 | Quadratic | 61403.13 | 61349.12 | 61295.51 | 0.00 | 84.82 |
|  | 4 | Cubic | 60917.16 | 60850.43 | 60784.21 | 4.19 | 85.67 |
|  | 5 | No time effect | 62804.36 | 62769.41 | 62734.72 | 0.00 | 88.73 |
|  | 5 | Linear | 61072.86 | 61022.02 | 60971.56 | 0.00 | 78.82 |
|  | 5 | Quadratic | 61436.46 | 61369.73 | 61303.51 | 0.00 | 84.15 |
|  | 5 | Cubic | 60961.27 | 60878.65 | 60796.66 | 0.00 | 82.80 |
|  | 6 | No time effect | 62821.02 | 62779.71 | 62738.72 | 0.00 | 87.52 |
|  | 6 | Linear | 61503.98 | 61443.60 | 61383.69 | 0.00 | 82.63 |
|  | 6 | Quadratic | 61003.84 | 60924.40 | 60845.56 | 0.00 | 82.53 |
|  | 6 | Cubic | 61006.30 | 60907.80 | 60810.04 | 0.00 | 83.23 |
| Externalising symptom models | 1 | No time effect | 69360.79 | 69351.26 | 69341.80 | 100 |  |
|  | 1 | Linear | 69153.45 | 69140.74 | 69128.12 | 100 |  |
|  | 1 | Quadratic | 69149.50 | 69133.61 | 69117.85 | 100 |  |
|  | 1 | Cubic | 69149.46 | 69130.40 | 69111.48 | 100 |  |
|  | 2 | No time effect | 69077.65 | 69061.76 | 69045.99 | 15.52 | 84.48 |
|  | 2 | Linear | 68543.38 | 68521.14 | 68499.07 | 8.36 | 91.64 |
|  | 2 | Quadratic | 68538.75 | 68510.15 | 68481.77 | 8.65 | 91.35 |
|  | 2 | Cubic | 68542.09 | 68507.14 | 68472.46 | 8.65 | 91.35 |
|  | 3 | No time effect | 69094.31 | 69072.06 | 69049.99 | 0.00 | 80.38 |
|  | 3 | Linear | 68292.92 | 68261.15 | 68229.62 | 6.12 | 82.21 |
|  | 3 | Quadratic | 68280.99 | 68239.68 | 68198.69 | 5.21 | 82.19 |
|  | **3** | **Cubic** | **68288.40** | **68237.56** | **68187.11** | **5.69** | **81.63** |
|  | 4 | No time effect | 69110.97 | 69082.37 | 69053.99 | 0.00 | 78.67 |
|  | 4 | Linear | 68317.91 | 68276.61 | 68235.62 | 0.00 | 78.45 |
|  | 4 | Quadratic | 68314.31 | 68260.29 | 68206.69 | 0.00 | 78.91 |
|  | 4 | Cubic | 68330.05 | 68263.32 | 68197.11 | 0.00 | 77.39 |
|  | 5 | No time effect | 69427.37 | 69392.42 | 69357.73 | 0.00 | 53.70 |
|  | 5 | Linear | 68342.91 | 68292.07 | 68241.62 | 0.00 | 78.28 |
|  | 5 | Quadratic | 68347.63 | 68280.90 | 68214.69 | 0.00 | 75.42 |
|  | 5 | Cubic | 68371.70 | 68289.09 | 68207.11 | 0.00 | 79.92 |
|  | 6 | No time effect | 69444.05 | 69402.74 | 69361.75 | 0.00 | 53.70 |
|  | 6 | Linear | 68367.90 | 68307.52 | 68247.62 | 0.00 | 71.63 |
|  | 6 | Quadratic | 68380.95 | 68301.52 | 68222.69 | 0.00 | 74.36 |
|  | 6 | Cubic | 68062.36 | 67963.86 | 67866.11 | 2.15 | 79.80 |

***Note.*** Model with the optimal number of trajectories is displayed in bold face.

‘Class’ refers to number of classes (trajectories) considered in each model. ‘% per class’ refers to percentage of participants in each class.

Min = Minimum; Max = Maximum; BIC = Bayesian information criterion; SABIC = Sample-adjusted Bayesian information criterion; AIC = Akaike information criterion.

**Table S3. Relationship between the symptom trajectory membership and covariates, for the non-normative trajectory groups only.**

|  | Internalising trajectory class | | | | Externalising trajectory class | | | |
| --- | --- | --- | --- | --- | --- | --- | --- | --- |
|  | *OR* | *LB (CI_95_)* | *UB (CI_95_)* | *Z* | *OR* | *LB (CI_95_)* | *UB (CI_95_)* | *Z* |
| Outdoor play | 1.13 | 0.98 | 1.18 | 5.05** | 1.05 | 0.95 | 1.09 | 1.94 |
| Deprivation index (ref.: 1st quintile) |  |  |  |  |  |  |  |  |
| 2nd quintile | 0.68 | 0.58 | 0.84 | -3.55** | 0.87 | 0.67 | 1.04 | -1.5 |
| 3rd quintile | 0.49 | 0.45 | 0.6 | -6.89** | 0.83 | 0.87 | 0.98 | -2.17* |
| 4th quintile | 0.4 | 0.41 | 0.49 | -8.96** | 0.83 | 0.68 | 0.99 | -2.02* |
| 5th quintile | 0.45 | 0.49 | 0.55 | -7.86** | 0.74 | 0.79 | 0.88 | -3.47** |
| Changes in deprivation quintile from sweep 2 to sweep3 | 1.3 | 0.2 | 1.55 | 2.92* | 1.02 | 0.48 | 1.18 | 0.19 |
| Ethnicity (ref.: White Caucasian) | 0.36 | 1.64 | 0.43 | -11.21** | 0.56 | 0.44 | 0.67 | -6.15** |
| Child sex (ref.: Male) | 1.53 | 0.84 | 1.7 | 7.76** | 1 | 0.88 | 1.1 | 0.04 |
| Maximum level of education of  at least one parent (ref.: Higher or above) |  |  |  |  |  |  |  |  |
| Standard grade or other | 0.82 | 0.99 | 0.92 | -3.21** | 0.73 | 0.97 | 0.82 | -5.36** |
| No qualifications | 0.92 | 0.69 | 1.06 | -1.11 | 0.88 | 1.12 | 1.01 | -1.88 |
| Parent working status (ref.: parents not working) | 1.22 | 0.55 | 1.39 | 2.96** | 1.92 | 0.82 | 2.17 | 10.19** |
| Respondent’s mental health | 1.21 | 0.73 | 1.26 | 9.51** |  |  |  |  |
| Access to a park within 10 minutes (ref.: No) | 0.81 | 0.94 | 0.95 | -2.59* | 1.19 | 1.05 | 1.24 | 8.8** |
| Access to a garden (ref.: No) | 1.09 | 0.16 | 1.26 | 1.23 | 1.04 | 0.69 | 1.2 | 0.54 |
| Child health problems | 0.94 | 1.65 | 0.97 | -3.75** | 1.45 | 0.48 | 1.65 | 5.49** |
| Symptom trajectory class of the opposed domain (ref.: normative)^1^ |  |  |  |  | 0.82 | 4.04 | 0.84 | -12.55** |
| Decreasing trajectory | 0.14 | 1.53 | 0.16 | -28.85** | 0.21 | 1.16 | 0.24 | -24.25** |
| Increasing trajectory | 0.34 | 1.53 | 0.38 | -17.29** | 1.39 | 1.16 | 1.66 | 3.48** |

*Note.* The outcome is the membership to an at-risk trajectory class of symptoms, with reference category = decreasing class membership (vs. the increasing class). This analysis just included individuals from the either decreasing class or increasing class membership. This regression model fitted better to data with Akaike Information Criterion (AIC) = 5270.42 (AIC for the unconstrained model = 5922.81), for the internalising symptom trajectory class outcome; and AIC = 7080.89 (AIC for the unconstrained model = 7815.2), for the internalising symptom trajectory class outcome. The model included the longitudinal weights for the cohort, as a weighting variable.

*OR* = Odds ratio. LB (CI_95_) = 95% Confidence interval of the OR (lower bound). UB (CI_95_) = 95% Confidence interval of the OR (upper bound). *Z* = Wald’s test statistic.

^1^ = The externalising trajectory class membership was used as a covariate for the internalising trajectory regression model. By contrast, the internalising trajectory class membership was used as a covariate for the externalising trajectory regression model.

* *p* < .05. ** *p* < .01.

**Figure S1.** Average trajectory of SDQ internalising (left side) and externalising (right side) symptoms across childhood measurement points.

**Figure S2.** Directed Acyclic Graph (DAG)

Directed Acyclic Graph created using DAGitty software. Green oval indicates exposure variable. Blue ovals with I indicate outcome variables. White ovals indicate variables that are adjusted for in the statistical models. Blue ovals indicate ancestor of exposure only. Pink ovals indicate un-adjusted ancestor of both exposure and outcome (confounding variable).

**Figure S3.** Density plot on the original distribution of variables (blue ink) with imputed data. The distribution of imputed data are displayed according to the different round of iterations (in red ink).

*Note*. class_ext = Externalising symptom trajectory class (only imputed for the internalising symptom trajectory class regression analysis). DaEthGpC = Ethnic group. MaHprb01 = Child health problems. MaZgar03_age2 = Access to a garden. parent_work_age2 = Parent working status. ALbSNimd = Deprivation index. McHGsx1 = Child sex. McApha08 = Access to a park within 10 minutes. DdWTbth2 = Birth cohort longitudinal weight. Play = Outdoor play (all sweeps in analysis). change_SIMD_SW2_3 = Change in Deprivation index from sweep 2 to sweep 3). max_ed_level_parent = Maximum level of education of at least one parent.

**Figure S4.** Proportion of sample belonging to each trajectory category across internalising and externalising domains.
